# Spatiotemporal patterns of aboveground vegetation and soil seed banks in desert steppe under photovoltaic disturbance

**DOI:** 10.3389/fpls.2026.2000199

**Published:** 2026-10-28

**Authors:** Qing Bai, Hao Zhang, Xiaomei Gong, Jianchu Xu, Qiang Yu, Changhui Peng, Xiaowei Li, Haiying Hu, Jianping Li, Fan Sang, Yingzhong Xie, Liang Guo

**Affiliations:** 1 School of Forestry and Grassland Science, Ningxia University, Yinchuan, Ningxia, China; 2 State Key Laboratory of Soil and Water Conservation and Desertification Control, College of Soil and Water Conservation Science and Engineering, Northwest A&F University, Yangling, Shaanxi, China; 3 Ningxia International Desertification Control Cooperation Center, Yinchuan, Ningxia, China; 4 Center for Mountain Ecosystem Studies, Kunming Institute of Botany, Chinese Academy of Sciences, Kunming, Yunnan, China; 5 World Agroforestry Center, Nairobi, Kenya; 6 School of Geographic Sciences, Hunan Normal University, Changsha, Hunan, China; 7 Department of Biology Science, Institute of Environment Sciences, University of Quebec at Montreal, Montreal, QC, Canada; 8 Administration Bureau of Ningxia Yunwushan National Nature Reserve, Guyuan, Ningxia, China; 9 Institute of Soil and Water Conservation, Chinese Academy of Sciences and Ministry of Water Resources, Yangling, Shaanxi, China

**Keywords:** functional traits, microhabitats, photovoltaic installations, plant communities, soil seed bank

## Abstract

The unique microenvironment created by photovoltaic (PV) power stations may progressively reshape ecosystem species composition through sustained effects on aboveground vegetation and soil seed banks. However, the long-term ecological impacts, particularly those on soil seed banks, remain poorly understood. We investigated aboveground vegetation and soil seed banks across three PV power stations with different operational durations (5, 10, and 15 years) in desert steppe ecosystems to assess how PV-induced microhabitats filter vegetation and seed bank assemblages. Our results show that with increasing operational duration, the density of soil seed bank outside the PV stations remained stable, whereas it increased progressively between panels, from 1657 seeds·m^-^² at 5 years to 2279 seeds·m^-^² at 10 years and 2880 seeds·m^-^² at 15 years, and declined significantly beneath panels. Across all operational durations, seed bank density consistently followed the spatial gradient: between panels > outside the PV stations > beneath panels. The spatial distribution of aboveground vegetation mirrored that of the seed bank and patterns of α-diversity exhibited similar trends. Significant differences in community species composition were observed among locations within each operational duration, and community differentiation intensified over time. Early-stage divergence was primarily driven by a few dominant species but gradually shifted toward regulation by multiple species. In addition, the functional composition of both the aboveground community and soil seed banks changed: the proportion of perennial prostrate taproot clonal plants increased beneath panels with operational duration, whereas annual prostrate taproot non-clonal plants increased significantly between panels. Overall, PV stations progressively restructure plant communities and soil seed banks by creating spatially heterogeneous microenvironments, with ecological effects intensifying over time. These findings provide valuable insights for the ecological management and sustainable land-use planning of PV installations in arid and semi-arid regions.

## 1 Introduction

The transition to low-carbon energy systems is a key strategy for reducing greenhouse gas emissions and achieving carbon neutrality (Emodi et al., 2019; Chu et al., 2024; Wang et al., 2025a). Solar photovoltaic (PV) energy has become an increasingly important component of this transition because of its technological maturity and potential for widespread deployment (Dinçer, 2011; Mitrašinović, 2021). Global PV installations expanded by 601 GW in 2024, bringing total installed capacity to more than 2.26 TW (IEA PVPS, 2025). As PV facilities expand into arid and semi-arid regions, their ecological effects are of particular concern in desert grasslands, where vegetation is sensitive to environmental disturbance (Xu et al., 2018). PV panels alter light availability and redistribute precipitation, creating distinct microhabitats beneath and between panels (Osman et al., 2023). Understanding how plant communities respond to these conditions is therefore important for assessing the ecological consequences of PV expansion in desert grasslands.

Environmental differences created by PV panels may influence plant communities through environmental filtering. Variation in resource availability can favor species with different functional traits and reproductive strategies, thereby altering community composition and diversity (Gámez-Virués et al., 2015; Oliveira et al., 2019). Studies of aboveground vegetation in PV facilities have nevertheless reported contrasting diversity responses, with some finding increases and others finding decreases (Cheng et al., 2026; Wang et al., 2025b). These contrasting findings suggest that the effects of PV installations may depend on the microhabitat examined and the species capable of establishing there. Although remote sensing provides valuable observations at large spatial scales, it is less suited to resolving fine-scale differences among PV microhabitats and cannot directly characterize belowground seed reserves (Hilker et al., 2024; Cui et al., 2025). Field observations comparing vegetation beneath panels, between panels, and in surrounding grasslands are therefore needed to clarify how PV-induced environmental heterogeneity shapes community assembly.

Aboveground vegetation alone, however, does not fully capture a plant community’s capacity for persistence and regeneration. Soil seed banks provide propagules for vegetation renewal following disturbance and constitute an important component of community resilience (Long et al., 2015; Bradbury et al., 2016; Yang et al., 2021; Li et al., 2025a). PV installations can alter soil environmental conditions (Zhang et al., 2023; Iranzo et al., 2025; Siggers et al., 2025), potentially affecting seed survival, germination, and persistence (Hoyle et al., 2013; Porto et al., 2018). Yet soil seed banks in PV facilities have received considerably less attention than aboveground vegetation (Vaverková et al., 2022; Liu et al., 2023). Li et al., 2025b), for example, reported lower seed bank richness and diversity in semi-arid grasslands with PV installations. How these responses vary among PV microhabitats, however, remains unclear. It is also uncertain whether changes in soil seed banks correspond to those in aboveground communities and whether this relationship varies with operational duration. Examining both components together is necessary to understand the potential consequences of PV development for vegetation regeneration.

Ningxia Hui Autonomous Region provides a setting for investigating these questions. The region has experienced severe desertification and substantial efforts to combat it (Guo et al., 2020; Huang et al., 2018; Liu et al., 2019a). Its abundant solar resources have supported the expansion of PV facilities since around 2010, with many installations established on desert grasslands under grazing exclusion management (Sun et al., 2021; Zhao et al., 2024). This development history provides an opportunity to examine plant communities across facilities with different operational durations. Comparisons among panel microhabitats, surrounding grasslands, and facilities of different ages can help determine whether community differences remain consistent or change as PV operation continues.

To address these questions, we investigated how PV-induced microenvironmental heterogeneity restructures aboveground plant communities and soil seed banks in desert grasslands and sought to identify the ecological mechanisms underlying these responses. We hypothesized that PV infrastructure reshapes community assembly by creating heterogeneous microhabitats and altering environmental filtering. Specifically, we proposed three hypotheses. (1) Reduced light availability and altered soil moisture beneath PV panels would strengthen environmental filtering and reduce species diversity, whereas relatively favorable inter-panel habitats would promote colonization and maintain diversity over time. (2) During the early stages of PV operation, community differentiation would be driven primarily by a limited number of stress-tolerant species. With prolonged operation, increasing habitat heterogeneity and declining dominance effects would allow a broader range of species to contribute to community divergence. (3) The distinct microhabitats beneath and between PV panels would select for species with particular life forms and growth traits. By integrating observations of aboveground vegetation and soil seed banks, this study aims to advance understanding of how renewable energy infrastructure influences community assembly and provide ecological guidance for reconciling renewable energy expansion with biodiversity conservation in desert grasslands.

## 2 Materials and methods

### 2.1 Study area

The study was conducted in the Taiyangshan Town (Wuzhong City, Ningxia Hui Autonomous Region, China), situated in the central arid zone of the upper Yellow River Basin. The region lies at an elevation of 1240–1450 m a.s.l. and is characterized by a typical temperate continental climate, with high potential evaporation, and abundant solar radiation (Xie et al., 2025). The soils in this region are predominantly aeolian sandy soils with coarse texture and low water-holding capacity, which contribute to high ecosystem sensitivity to environmental change (Tian et al., 2021). The natural vegetation is dominated by xerophytic plant communities adapted to chronic water limitation. Dominant species include *Stipa breviflora*, *Salsola collina*, and *Lespedeza potaninii*, which collectively constitute the structural foundation of the regional desert steppe ecosystem (Yu et al., 2022; Chen et al., 2024). This area represents one of the earliest and largest solar energy development clusters in Ningxia. Large-scale PV construction began in 2010, followed by continuous increases in installed capacity and land occupation (Xiao et al., 2025). The progressive expansion of PV facilities has generated a chrono sequence of sites differing in operational durations while experiencing broadly comparable climatic and geomorphological conditions. This provides an ideal natural experiment for examining how PV infrastructure influences aboveground vegetation and soil seed bank characteristics across different stages of ecosystem development.

### 2.2 Experimental design

Three PV sites with operational durations of 5 years (37°49′48.96″N 106°58′18.64″E), 10 years (37°48′7.08″N 106°53′53.41″E), and 15 years (37°48′40.05″N 106°53′34.36″E) were selected for this study. All PV systems were fixed-tilt installations with comparable structural configurations. Panel heights and spacing were similar among sites: at the 5-year site, panels were positioned 0.8– 2.6 m above ground with a spacing of 5.8 m; at the 10-year site, 0.3–2.2 m above ground with a spacing of 5.0 m; and at the 15-year site, 0.8–2.8 m above ground with a spacing of 6.0 m. At each site, aboveground vegetation surveys and soil seed bank sampling were conducted using quadrats established in three microhabitats: outside the panels (Outside; located within the PV facility but not directly covered by panels), between panels (Between), and beneath panels (Beneath) (**Figure 1a**). All PV sites were located on relatively flat desert grasslands and enclosed by fences to minimize grazing and other anthropogenic disturbances. Each site covered more than 2 km². To minimize edge effects, all sampling locations were established within the interior of each PV site, well away from perimeter fences and access roads. To verify climatic comparability among the three PV sites, monthly air temperature and precipitation data (1901–2024) were extracted from the CRU TS v4.09 dataset based on site coordinates (Peng et al., 2017; Peng, 2020). The three sites exhibited highly consistent climatic conditions throughout the entire period, particularly during the 15-year study period (**Supplementary Figure S1**). The three sites were located within a distance of 0.7–4.4 km, with pairwise correlations exceeding 0.999 for both temperature and precipitation. Moreover, site identity explained only 0.43% of the variation in temperature and 0.59% of the variation in precipitation (**Supplementary Figure S2**), confirming that the three sites experienced nearly identical climatic conditions.

**Figure 1:**
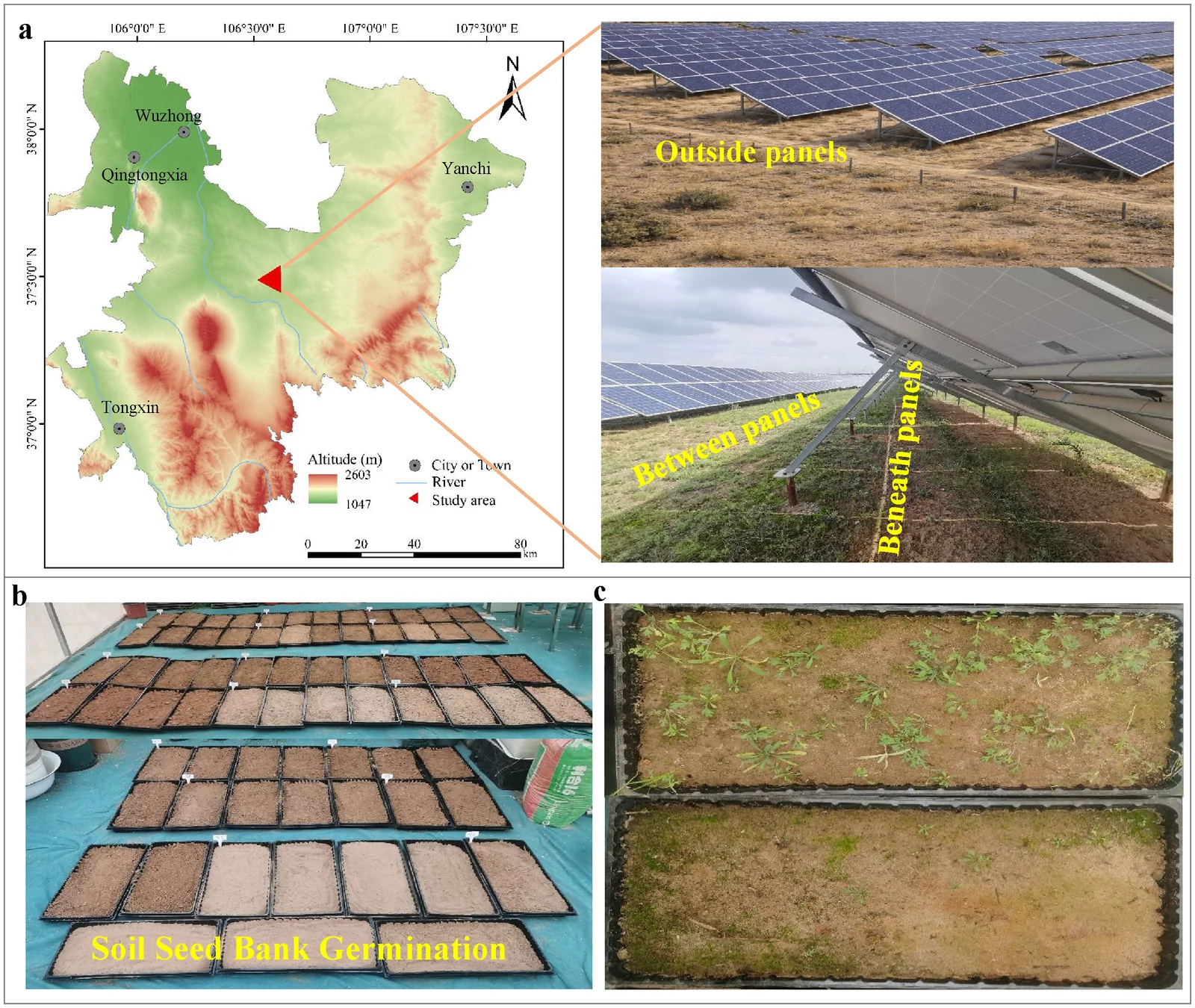
Study sites and sampling locations **(a)**, and photographs of the soil seed bank germination experiment **(b, c)**.

### 2.3 Aboveground plant community survey and seed bank sampling and germination

#### 2.3.1 Aboveground vegetation survey and soil microclimate measurements

To characterize plant community structure, an aboveground vegetation survey was conducted in late August 2024, corresponding to the peak growing season in the study area. At each PV site representing 5-, 10-, and 15-year operational stages, three typical microhabitats were investigated. Within each microhabitat, four 50 cm × 50 cm quadrats were randomly established and spatially distributed to capture within-habitat variability. In each quadrat, all plant species were identified and their abundances recorded. Species abundance and richness were subsequently calculated at the quadrat level. For the beneath-panel microhabitat, quadrats were positioned at the center of the panel projection area to minimize edge effects caused by spatial variation in light interception and runoff redistribution. In total, 36 vegetation quadrats were surveyed across the three PV sites. In addition, soil temperature (0–10 cm depth) was measured *in situ* using a portable soil temperature logger (RD1200, ACCLIMA, USA), and gravimetric soil water content was determined by oven-drying soil samples at 105 °C to constant mass (Reynolds, 1970).

#### 2.3.2 Soil seed bank sampling and germination

Concurrent with the aboveground vegetation survey, four soil cores (10 cm diameter) were randomly collected from each vegetation quadrat for soil seed bank analysis. Each core was divided into two depth intervals (0–5 cm and 5–10 cm), and samples from the same quadrat and depth were pooled into a composite sample to account for microscale spatial heterogeneity. This sampling design yielded 24 seed bank samples for each PV operational duration (5, 10, and 15 years), resulting in 72 composite samples in total. Then all soil samples were air-dried under well-ventilated conditions and sieved through a 4 mm mesh to remove stones and plant debris. The processed samples were subsequently stored under dry and well-ventilated conditions until germination experiments.

In October 2024, a greenhouse germination experiment was conducted to quantify soil seed bank composition and abundance. Standardized plastic seedling trays (45 cm length, 30 cm width, and 7 cm height) were used as germination units. A 3-cm layer of fine sand was placed at the bottom of each tray to facilitate drainage and provide a seed-free substrate. The sand was oven-dried at 105 °C for 24 h to eliminate any potential seed contamination. Each soil sample was evenly spread over the sand layer to a depth of approximately 2–3 cm. All trays were maintained in a greenhouse at the National University Science and Technology Park of Ningxia University under controlled temperature (15–25 °C) and regularly watered to maintain suitable moisture conditions for seedling emergence. Seedlings were monitored twice weekly. Once seedlings reached a stage suitable for morphological identification, they were identified, counted, and removed to avoid interference with subsequent germination events. Germination was considered complete when no new seedlings emerged for four consecutive weeks (**Figures 1b, c**). The germination experiment continued until mid-March 2025, providing an approximately five-month observation period to characterize seedling emergence dynamics under standardized temperature and moisture conditions while minimizing the influence of seasonal constraints in field environments.

### 2.4 Data analysis

To systematically assess the diversity of aboveground plant communities and soil seed banks, three commonly used diversity indices were calculated: the Simpson diversity index (*D*), the Shannon-Wiener diversity index (*H*), and the Margalef richness index (*R*) (Supriatna, 2018). Differences in α-diversity, species abundance, and soil seed bank density were tested using one-way analysis of variance (ANOVA). Specifically, analyses examined spatial variation among microhabitats within each PV operational duration and temporal variation among operational durations within each microhabitat.


$$D=1-\sum_{i=1}^{s}P_{i}^{2}$$

$$H=-\sum_{i=1}^{s}P_{i}\times \ln P_{i}$$

R=(S−1)/lnN

Where: *P_i_* represents the relative abundance of species *i*, calculated as the proportion of individuals of species *i* relative to the total number of individuals in a quadrat; *S* is the total number of species in the aboveground plant community or soil seed bank; and *N* is the total number of individuals recorded in the corresponding quadrat.

To assess β diversity patterns in community composition for both aboveground vegetation and soil seed banks, non-metric multidimensional scaling (NMDS) based on Bray-Curtis distance matrices was performed to visualize compositional similarities among samples (Byrnes et al., 2014). Permutation-based multivariate analysis of variance (PERMANOVA) was subsequently used to test the statistical significance of differences in community composition among groups. Additionally, similarity percentage analysis (SIMPER) was employed to identify the species contributing most to the observed inter-group dissimilarities (Gioria and Osborne, 2009).

To identify functional groups filtered by PV infrastructure, hierarchical clustering analysis was performed using six plant reproductive-related plant functional traits (**Supplementary Table S1**). Since the trait dataset included both numerical and categorical variables, Gower’s distance was first used to construct a dissimilarity matrix (Vineetha and Nandan, 2022). Hierarchical clustering was then conducted using Ward’s minimum variance method, which produces clusters with high internal similarity (Hu et al., 2000). The optimal number of clusters was determined using the silhouette coefficient, which evaluates clustering quality by comparing within-cluster similarity to between-cluster dissimilarity (Massucato et al., 2019). Based on these results, plant functional groups were subsequently delineated. All statistical analyses and visualizations were performed in R version 4.5.0, primarily using the packages *vegan*, *ggpubr*, *phyloseq*, and *cluster*.

## 3 Result

### 3.1 Spatiotemporal heterogeneity in abundance of aboveground vegetation and soil seed banks across PV microhabitats

A total of 35 plant species from 14 families and 31 genera were recorded in the aboveground vegetation survey, whereas the soil seed bank contained 39 species from 15 families and 34 genera (**Supplementary Table S2**). Seed bank density exhibited a pronounced vertical pattern, with the 0–5 cm soil layer containing significantly higher densities than the 5–10 cm layer and accounting for more than 85% of total seed abundance (**Supplementary Table S3**). Notably, nearly all species detected in the deeper layer were also present in the upper layer. Therefore, the two soil layers were pooled for subsequent analyses.

Across sites with the same operational duration, seed bank density consistently followed the spatial gradient: between panels (Between) > outside the PV power station (Outside) > beneath panels (Beneath). In the Beneath microhabitat, the relative abundance of Poaceae was significantly lower than that in the Between and Outside microhabitats, whereas Asteraceae showed the opposite pattern (**Figure 2**). These shifts were mainly associated with changes in the relative abundance of the genera *Stipa* and *Artemisia* (**Supplementary Figure S3**). In aboveground vegetation, Fabaceae consistently exhibited the highest relative abundance beneath panels across all operational durations (**Figures 2a, b, c**). Notably, Poaceae showed similar relative abundance patterns between aboveground vegetation and soil seed banks, indicating partial concordance between aboveground communities and belowground propagule pools (**Figure 2**). With increasing PV operational duration, seed bank density between panels increased from 1657 and 2279 seeds·m^-^² at 5 and 10 years, respectively, to 2880 seeds·m^-^² after 15 years (**Supplementary Table S4**). In contrast, seed bank density beneath panels decreased over time, whereas density outside PV facilities showed relatively little temporal variation. Further analysis at the genus level confirmed the consistency of these patterns, with aboveground vegetation and soil seed banks displaying similar compositional trajectories across PV operational durations (**Supplementary Figures S3**, **S4**).

**Figure 2:**
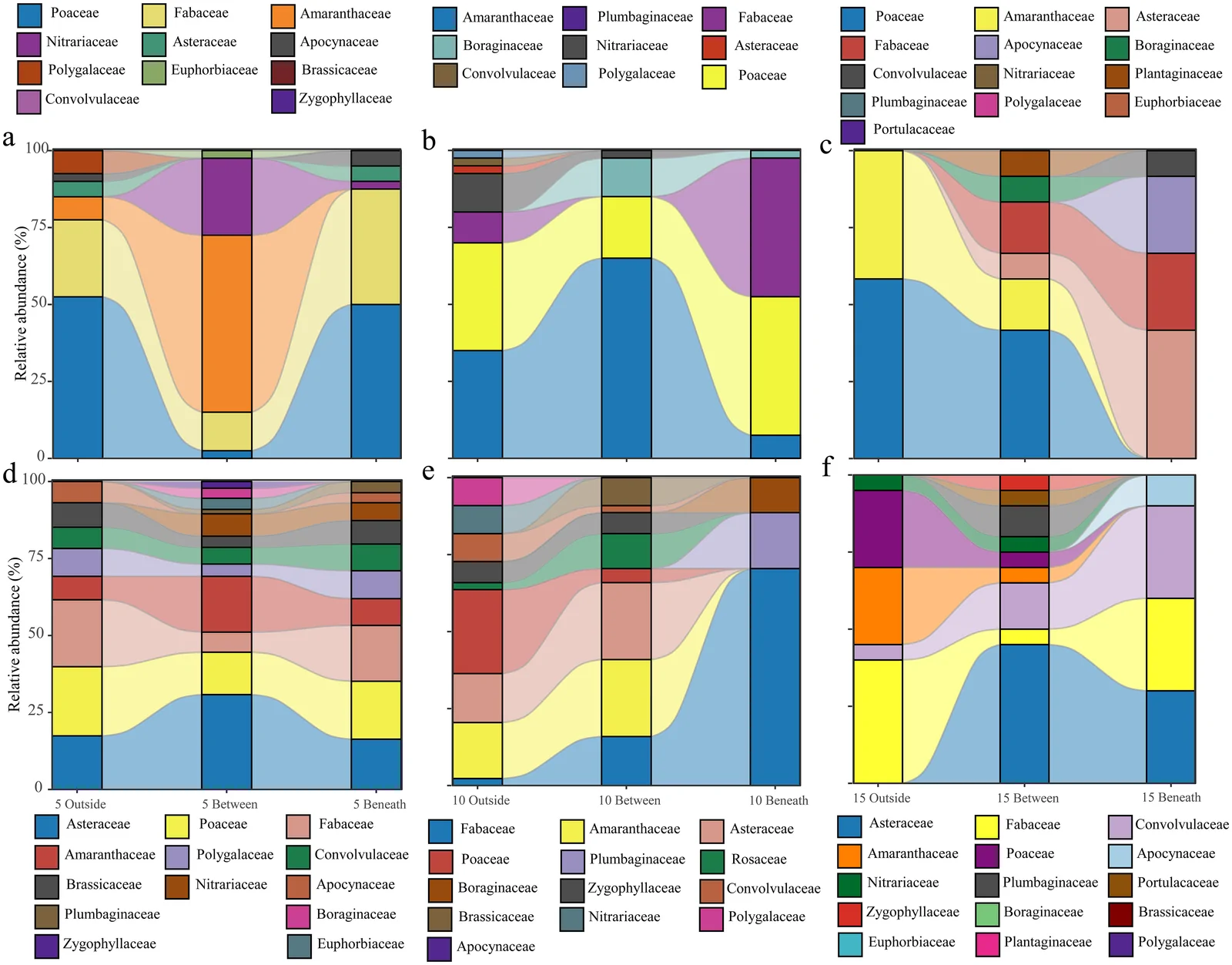
Relative changes in family-level species richness of aboveground vegetation **(a-c)** and soil seed banks **(d-f)** across different microhabitats within photovoltaic (PV) power stations of different operational durations. “Outside” indicates areas outside the PV field, while “Between” and “Beneath” denote locations between and beneath PV panels, respectively. The numbers 5, 10, and 15 represent PV operational durations in years. The same notation applies to subsequent figures.

### 3.2 Spatiotemporal heterogeneity in the diversity of aboveground vegetation and soil seed banks across PV microhabitats

Except for the soil seed bank α-diversity index at the 5-year site and the Margalef richness index of aboveground vegetation, α-diversity generally exhibited a consistent spatial pattern within each operational duration, with the highest values occurring between panels and the lowest beneath panels (**Figure 3**). Notably, although the Simpson index of aboveground vegetation beneath panels significantly decreased after 5 years of PV operation, no corresponding change was observed in the soil seed bank community (**Figures 3a, d**). In addition, changes in the Simpson index of the soil seed bank occurred more slowly than those in the Shannon diversity index (**Figures 3d–f**). Across operational stages, diversity response at the same microhabitat showed contrasting patterns: α-diversity index of both aboveground vegetation and soil seed banks increased between panels but decreased beneath panels with increasing PV operational duration.

**Figure 3:**
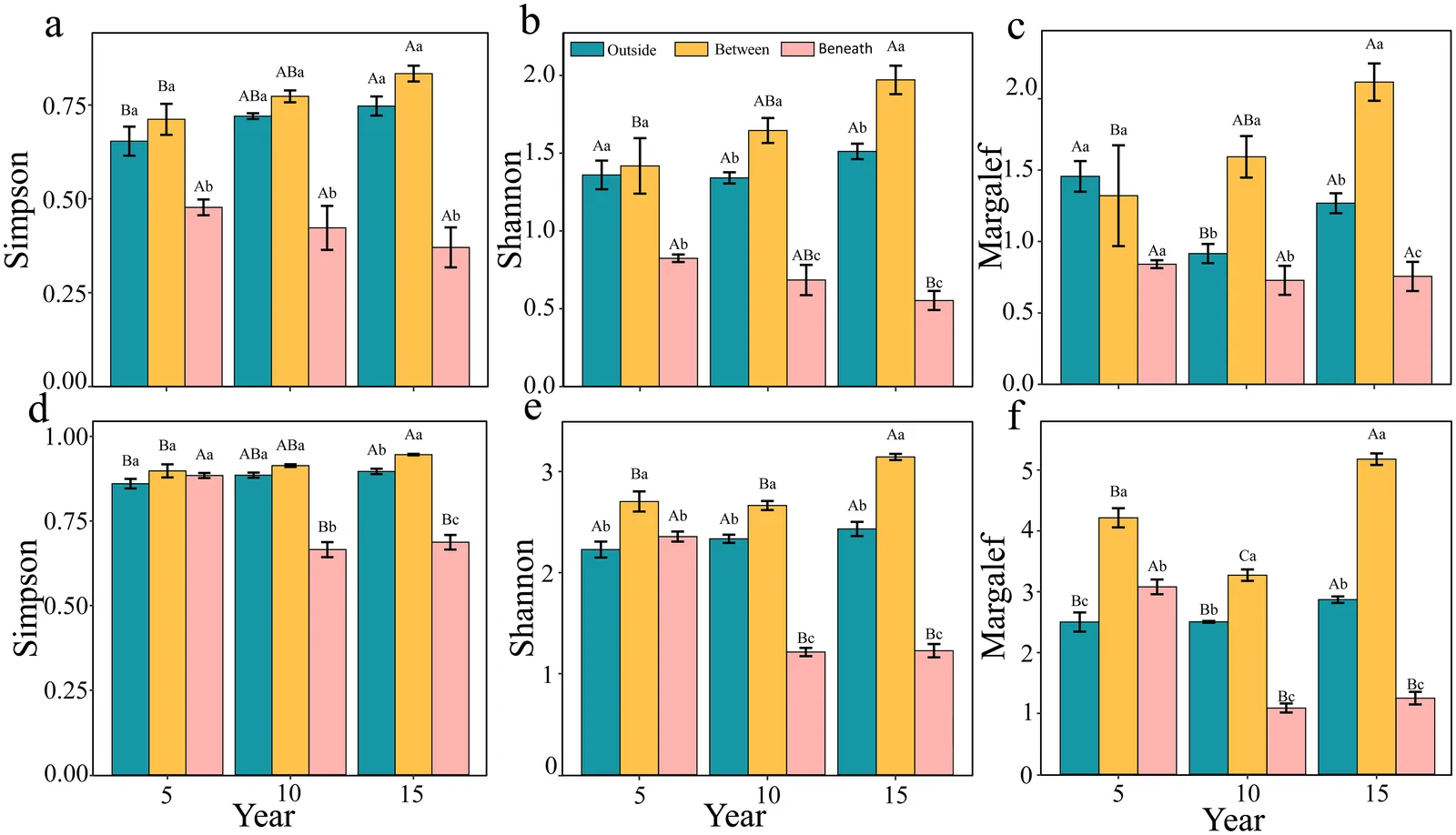
Effects of photovoltaic (PV) microhabitats on the α-diversity of aboveground vegetation **(a-c)** and soil seed banks **(d-f)**. Different lowercase letters indicate significant differences among microhabitats within the same PV operational duration, whereas different uppercase letters indicate significant differences among operational durations within the same microhabitat.

### 3.3 Differences in community composition of aboveground vegetation and soil seed banks across PV microhabitats

NMDS and PERMANOVA analyses revealed significant differences in species composition of both aboveground vegetation and soil seed banks among PV microhabitats (Outside, Between, Beneath) within each operational duration (*P* < 0.01; **Figure 4**; **Supplementary Figure S5**). The ordination patterns showed clear separation among microhabitats, with particularly strong differentiation along the NMDS1 axis for soil seed banks, where all three microhabitats were distinctly separated. SIMPER analysis further identified the species contributing most to these compositional differences. In aboveground vegetation, the major contributors varied among operational stages, with *Stipa breviflora* dominating at the 5-year site, *Grubovia dasyphylla* at the 10-year site, and *Leymus secalinus* at the 15-year site (**Supplementary Figure S5 b, d, f**). In contrast, for the soil seed bank, *Artemisia scoparia* was the main contributor at the 5-year and 15-year sites, whereas *Artemisia stelleriana* made the largest contribution at the 10-year site (**Figure 4b, d, f**).

**Figure 4:**
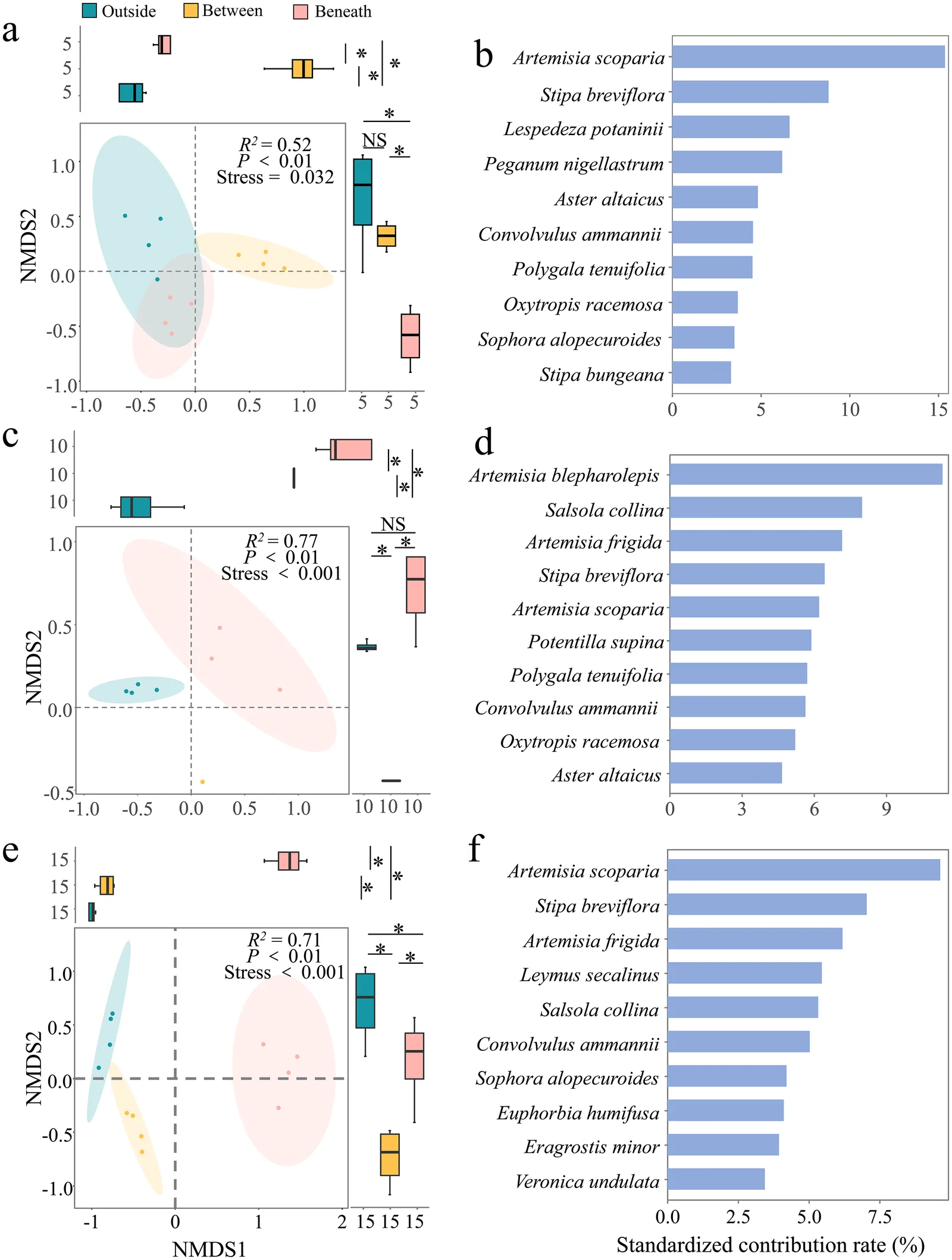
NMDS ordination **(a, c, e)** and SIMPER contribution analysis **(b, d, f)** of soil seed banks composition across different photovoltaic (PV) microhabitats. Asterisks (*) indicate significant differences between groups along the NMDS axes.

Notably, both aboveground vegetation and soil seed banks showed a gradual decline in the standardized species contribution of dominant species to community differentiation with increasing PV operational duration (**Figures 4b, d, f**; **Supplementary Figure S5b, d, f**). This indicates a temporal shift from dominance-driven community divergence toward more distributed species contributions as PV operation progressed.

### 3.4 Functional group patterns of aboveground vegetation and soil seed banks across PV microhabitats

Cluster analysis based on six plant functional traits (**Supplementary Table S1**) revealed that the silhouette coefficient reached its maximum value (0.52) when the number of clusters was set to five. Accordingly, the 39 plant species recorded in this study (**Supplementary Table S2**) were classified into five functional groups: 1) PETN, perennial erect and rosette taproot non-clonal; 2) PEFN, perennial erect fibrous root non-clonal; 3) PPTC, perennial prostrate taproot clonal; 4) APTN, annual prostrate taproot non-clonal; and 5) AETN, annual erect taproot non-clonal (**Figure 5**).

**Figure 5:**
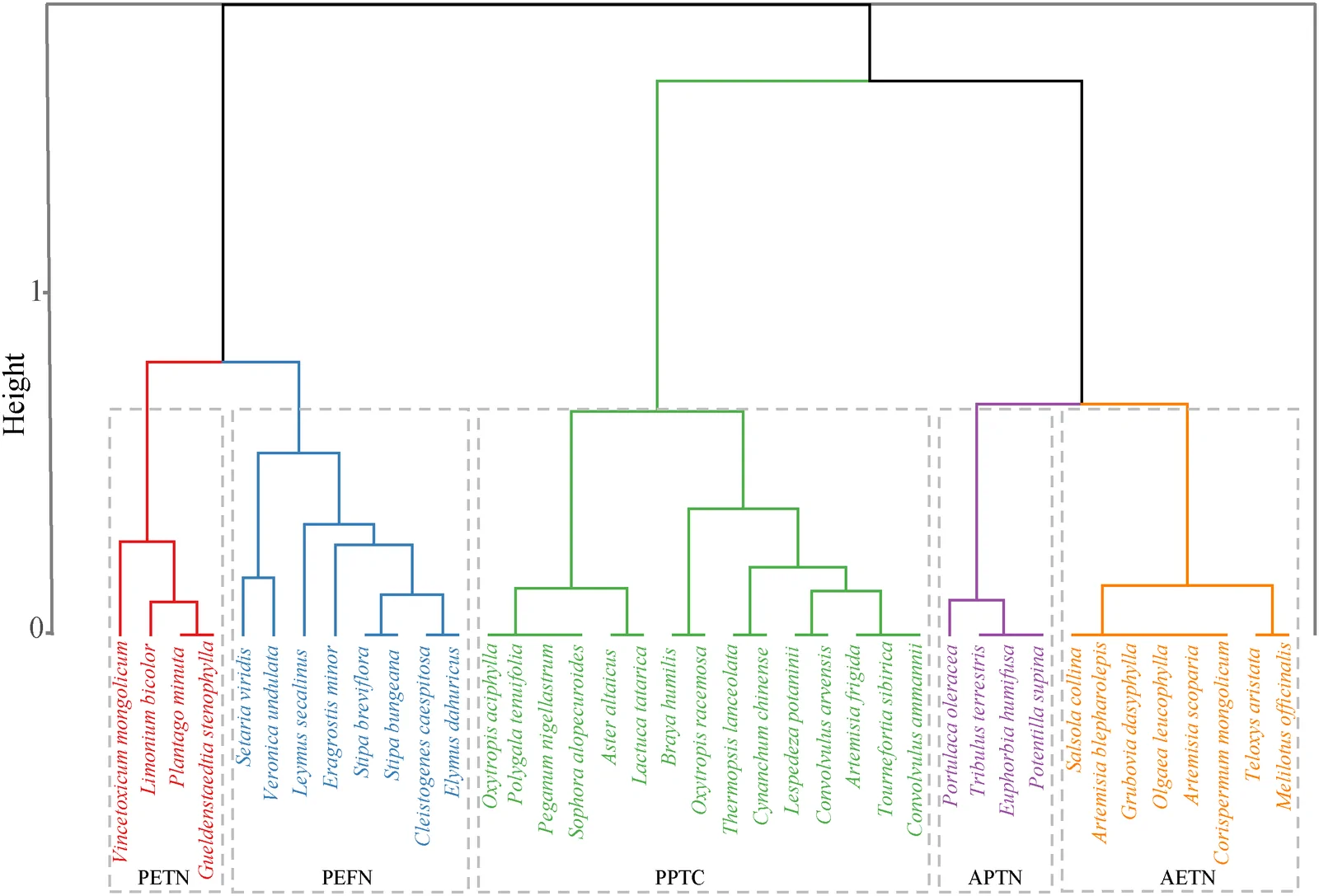
Cluster analysis of plant species based on six functional traits. The functional groups are defined as follows: PETN, perennial erect and rosette taproot non-clonal; PEFN, perennial erect fibrous root non-clonal; PPTC, perennial prostrate taproot clonal; APTN, annual prostrate taproot non-clonal; and AETN, annual erect taproot non-clonal.

Analysis of plant functional group composition in aboveground vegetation and soil seed banks revealed pronounced shifts across PV microhabitats with increasing operational duration. The proportion of PPTC species beneath panels increased progressively over time, whereas annual species between panels showed a significant increase (**Table 1**). At the 5-year sites, PPTC proportions did not differ significantly among the three microhabitats. However, at the 10- and 15-year sites, PPTC species exhibited significantly higher proportions beneath panels than in the Between and Outside microhabitats. Moreover, their proportion of PPTC species beneath panels increased steadily with PV operational duration, reaching 100% at the 15-year site.

**Table 1:** Proportional distribution of plant functional groups in soil seed bank and aboveground vegetation across different PV microhabitats.

Type	Group	PETN	PEFN	PPTC	APTN	AETN
Seed bank	5 Outside	0.054 ± 0.076 Aa	0.233 ± 0.148 Aa	0.577 ± 0.133 Aa	0 Bb	0.136 ± 0.057 Bb
	5 Between	0.029 ± 0.017 Ba	0.169 ± 0.062 Ba	0.395 ± 0.071 ABa	0.056 ± 0.03 Ba	0.351 ± 0.085 Aa
	5 Beneath	0.072 ± 0.097 ABa	0.183 ± 0.123 Aa	0.568 ± 0.152 Ba	0.006 ± 0.012 Ab	0.171 ± 0.059 Ab
	10 Outside	0 Ab	0.166 ± 0.053 Aa	0.556 ± 0.082 Ab	0.083 ± 0.049 Aa	0.195 ± 0.079 ABb
	10 Between	0.011 ± 0.011 Bb	0.085 ± 0.036 Cb	0.424 ± 0.031 Ab	0.127 ± 0.039 Aa	0.353 ± 0.046 Aa
	10 Beneath	0.178 ± 0.158 Aa	0 Bc	0.822 ± 0.158 Aa	0 Ab	0 Bc
	15 Outside	0.031 ± 0.014 Ab	0.301 ± 0.081 Aa	0.351 ± 0.113 Bb	0.06 ± 0.059 ABab	0.257 ± 0.073 Aa
	15 Between	0.111 ± 0.035 Aa	0.251 ± 0.028 Aa	0.318 ± 0.049 Bb	0.088 ± 0.026 ABa	0.232 ± 0.07 Ba
	15 Beneath	0 Bb	0 Bb	1 ± 0 Aa	0 Ab	0 Bb
Plant community	5 Outside	0.036 ± 0.055 Aa	0.472 ± 0.168 Aa	0.370 ± 0.249 Aa	0 Aa	0.122 ± 0.158 Bb
	5 Between	0 Aa	0.034 ± 0.048 Bb	0.423 ± 0.115 Aa	0.053 ± 0.065 ABa	0.490 ± 0.094 Aa
	5 Beneath	0.036 ± 0.071 Aa	0.500 ± 0.337 Aa	0.414 ± 0.310 Ba	0 Aa	0.050 ± 0.058 Ab
	10 Outside	0 Aa	0.403 ± 0.135 Aa	0.280 ± 0.095 ABb	0 A	0.317 ± 0.040 ABb
	10 Between	0.003 ± 0.006 Aa	0.275 ± 0.191 Aa	0.120 ± 0.107 Bc	0 B	0.601 ± 0.215 Aa
	10 Beneath	0 Aa	0.434 ± 0.031 Aa	0.496 ± 0.081 Ba	0 A	0.069 ± 0.094 Ac
	15 Outside	0 Aa	0.489 ± 0.179 Aa	0.035 ± 0.027 Bc	0.003 ± 0.006 Ab	0.472 ± 0.176 Aa
	15 Between	0.058 ± 0.061 Aa	0.364 ± 0.084 Aa	0.327 ± 0.122 Ab	0.070 ± 0.024 Aa	0.180 ± 0.085 Bb
	15 Beneath	0 Aa	0 Bb	1 ± 0 Aa	0 Ab	0 Ab

Different lowercase letters indicate significant differences among microhabitats within the same PV operational duration, whereas different uppercase letters indicate significant differences among operational durations within the same microhabitat.

## 4 Discussion

### 4.1 Effects of PV installations on the abundance and diversity of aboveground vegetation and soil seed banks

Our results reveal contrasting patterns of soil seed bank density and plant diversity among PV microhabitats in desert grasslands. Seed bank density was consistently higher between panels than in the Outside and Beneath microhabitats, following the order Between > Outside > Beneath at each facility (**Supplementary Table S4**). Between-panel density was 1657, 2279, and 2880 seeds·m^-^² at the 5-, 10-, and 15-year sites, respectively, with the value at the 15-year site approximately 73.8% higher than that at the 5-year site. Similarly, α-diversity of both aboveground vegetation and soil seed banks increased between panels with operational duration, whereas seed bank density and α-diversity declined beneath panels (**Figure 3**). These patterns likely reflect interactions among microenvironmental conditions, including light availability and soil moisture, as well as seed input, competition, and plant life histories. Because water availability is the primary limiting factor in desert grassland ecosystems, increased water inputs may alleviate drought stress (Na et al., 2019; Wang et al., 2026). PV panels redistribute precipitation and may create zones of greater water availability between panels (Chen et al., 2025), where exposure to direct sunlight is also higher. Beneath panels, rainfall interception occurs alongside shading. Although shading reduces light availability, it may also lower temperature and evaporative demand (Liu et al., 2019b). Its net effect therefore depends on the balance between water and light availability. In this study, soil moisture was lower beneath panels than between panels (**Supplementary Figure S6**), suggesting that any reduction in evaporation associated with shading was insufficient to offset this difference. Lower seed bank density and α-diversity beneath panels may thus reflect constraints on reproduction and establishment imposed by the combined limitation of light and water.

Seed bank density depends on the balance between seed inputs and losses through germination, mortality, and removal. Higher seed bank density between panels may result from increased local seed production, greater seed deposition from other locations, or enhanced seed persistence, without necessarily indicating improved seedling establishment (Huang et al., 2024). PV panels may alter near-surface airflow and runoff, potentially affecting the deposition of wind- and water-dispersed seeds (Armstrong et al., 2016) and creating heterogeneous seed rain patterns among the Beneath, Between, and Outside microhabitats. Changes in resource availability may also influence competition. Greater water and light availability between panels may promote the growth of established plants and intensify competition, while disturbance and habitat heterogeneity may create opportunities for species that colonize readily and depend on seed reproduction. Higher diversity between panels may therefore reflect the combined effects of increased seed supply, more opportunities for establishment, and competitive release.

Plant life histories further influence these processes. Annual plants depend on seed production and seedling recruitment to maintain local populations, whereas perennial and clonal plants can persist through the survival of established individuals or vegetative growth. Higher α-diversity between panels may therefore be associated with successful reproduction and recruitment of annual species under more favorable water and light conditions. Conversely, declining seed bank density beneath panels may reflect constraints on annual plant reproduction and recruitment under limited light and water availability, while perennial clonal species persist aboveground. These life-history differences may also help explain why aboveground vegetation and soil seed banks respond differently to the same habitat conditions.

The Simpson diversity index primarily reflects species dominance within a community, whereas the Shannon diversity index is more sensitive to changes involving less abundant species (Lozupone and Knight, 2008; Roswell et al., 2021; Mi et al., 2024). Within each operational duration, variation in the Simpson index among microhabitats was generally weaker than that in the Shannon index (**Figures 3a, b, d, e**). For example, at the 5-year PV site, soil seed bank diversity did not differ significantly among microhabitats according to the Simpson index, whereas the Shannon index indicated significantly higher diversity between panels than beneath panels or outside the PV installations. This discrepancy suggests that dominant species may remain relatively stable under PV-induced environmental changes, while rare species may respond more rapidly to environmental filtering by persisting under altered conditions or being excluded from the community.

Overall, these findings support the predictions of our first hypothesis regarding differences in diversity among microhabitats. The underlying mechanisms, however, likely involve interactions among resource filtering, seed supply, competition, and life-history strategies. Future studies could combine direct measurements of light availability, soil moisture dynamics, seed rain, and seedling recruitment with seed addition and neighbor removal experiments to test the pathways through which PV installations affect community diversity.

### 4.2 Key species contributions and aboveground-belowground community responses to PV installations

Microenvironmental changes can influence the structure of aboveground vegetation and soil seed banks, but their responses may differ in both rate and direction (Busso and Bonvissuto, 2009; Blonder et al., 2018). PERMANOVA analysis revealed significant spatial differences in community composition among the three PV microhabitats, and these differences persisted across operational durations. Although microhabitat location explained substantial variation in both aboveground vegetation and soil seed bank composition, the R² values were consistently higher for soil seed banks than for aboveground vegetation at the 10-year and 15-year sites (e.g., 10 years: 0.77 vs. 0.76; 15 years: 0.71 vs. 0.55) (**Figure 4**, **Supplementary Figure S5**). This suggests that community differentiation signals were more strongly retained in soil seed banks than in aboveground communities, likely reflecting the cumulative and persistent effects of PV-induced habitat modification. Thus, soil seed banks can function as a “historical memory” of plant community dynamics. Similar patterns have been reported by Carter Wynne and Sullivan (2025), who compared soil seed bank and aboveground vegetation responses under different restoration treatments.

SIMPER analysis further revealed a clear decoupling in the key species contributing to community differentiation between aboveground and underground components. Differences in soil seed bank composition at 5- and 15-year site were primarily driven by *Artemisia scoparia*, whereas *Artemisia blepharolepis* was the dominant contributor at the 10-year site. These key contributors differed from those identified in aboveground vegetation (*Stipa breviflora*, *Grubovia dasyphylla*, *Leymus secalinus*). This mismatch suggests that aboveground vegetation and soil seed banks may respond to similar environmental filters but with temporal lags and distinct ecological strategies. *Artemisia* species often contribute substantially to soil seed banks through abundant and persistent seed production (Li et al., 2011), whereas perennial grasses maintain aboveground dominance through long-lived growth forms and competitive persistence (Grime, 1977). Such asynchronous responses between aboveground and underground components may contribute to community resilience and facilitate dynamic stability under heterogeneous environmental conditions.

Importantly, the contribution of the dominant species to community differentiation declined with increasing PV operational duration. This pattern supports our second hypothesis, suggesting that early stages of community composition was primarily driven by a limited number of highly abundant dominant species (e.g., *Stipa breviflora*). With prolonged PV operation, increasing microenvironmental heterogeneity may facilitateniches differentiation, resulting in community divergence being increasingly shaped by a broader suite of species (e.g., *Grubovia dasyphylla*, *Leymus secalinus*, *Salsola collina*). These findings highlight the importance of adopting a dynamic perspective that integrates both aboveground and underground components when evaluating and managing artificial ecosystems such as PV installations.

### 4.3 Functional trait selection of plant communities under PV-induced microenvironmental heterogeneity

Trait-based clustering and functional group analyses revealed distinct responses of plant functional groups across the Beneath, Between, and Outside microhabitats of PV installations. The relative proportion of the PPTC functional group beneath panels increased with operational duration, reaching 100% at the 15-year site (**Table 1**). This pattern suggests that shaded, drought-stressed conditions beneath panels progressively favor perennial prostrate taproot clonal species with greater capacities for resource integration and spatial expansion (Keep et al., 2021; Yan et al., 2025). Prostrate growth keeps plants close to the soil surface, reducing water loss through transpiration (Wolf and Lundholm, 2008). Clonal reproduction and perennial life histories also allow these species to maintain populations without depending on annual recruitment, facilitating persistence under prolonged resource limitation. Conversely, the proportion of annual plants, particularly erect, non-clonal species, declined beneath panels with operational duration, suggesting that these species cannot persist under sustained resource limitation (Levine et al., 2010; Li et al., 2024).

Between-panel microhabitats appear to favor annual species because of greater resource availability (Bai et al., 2022; **Table 1**). APTN species consistently accounted for a high proportion of the soil seed bank between panels, suggesting that they can respond flexibly to environmental variation and maintain regeneration potential through persistent seed banks (Salguero‐Gómez, 2017). These patterns highlight the ecological importance of between-panel habitats as refugia and recruitment hotspots for short-lived species that depend on seed reproduction, thereby contributing to the maintenance of community diversity. These findings suggest that ecological management of PV installations should consider functional diversity alongside species richness. Beneath panels, promoting the establishment and persistence of perennial prostrate species with clonal growth and tolerance to shade and drought may improve vegetation cover and soil conservation. Between panels, conserving annual species and their soil seed banks is essential for maintaining regeneration potential and supporting long-term community succession.

### 4.4 Limitations and implications for ecological management of PV facilities

As photovoltaic development rapidly expands in dryland ecosystems, our study provides timely insights into the responses of aboveground vegetation and soil seed banks across different PV microhabitats and operational durations, with important implications for biodiversity conservation and ecological management. However, several limitations should be considered when interpreting and extrapolating our findings.

First, the structural parameters of the PV installations differed among the three facilities, and safety and access restrictions limited microclimatic measurements to soil moisture alone, so the microenvironment beneath and between panels could not be fully characterized. Second, comparisons among facilities with different operational durations cannot fully distinguish temporal effects from differences in PV configuration and site-specific conditions. Thus, the observed differences should be interpreted as associations across sites rather than direct evidence of long-term temporal changes. These limitations also constrain the broader applicability of our findings, which are most relevant to arid and semi-arid desert grassland PV systems with comparable climatic, edaphic, vegetation, and management conditions. Extrapolation to other ecosystems requires caution, as ecological responses may differ with PV design, array density, local environmental conditions, and land management practices.

Despite these limitations, the contrasting responses of aboveground vegetation and soil seed banks in our study offer practical implications for biodiversity conservation and ecological restoration in PV facilities. Specifically, beneath-panel, inter-panel, and surrounding grassland microhabitats should be considered separately when developing management strategies: beneath-panel areas may require particular attention to native species persistence and potential seed bank limitations, whereas inter-panel areas may offer opportunities to maintain native herbaceous vegetation and support natural regeneration. Surrounding grasslands can serve as reference communities for evaluating vegetation changes and may provide potential sources of native seeds. Joint assessments of aboveground vegetation and soil seed banks could further help identify areas where natural regeneration may be sufficient and where supplementary sowing of native species should be considered (**Figure 6**). Future research should combine standardized, replicated PV configurations with direct microclimatic measurements and long-term monitoring to clarify the underlying mechanisms and temporal dynamics. Such efforts would help refine site-specific restoration strategies and support adaptive management of desert grassland PV ecosystems.

**Figure 6:**
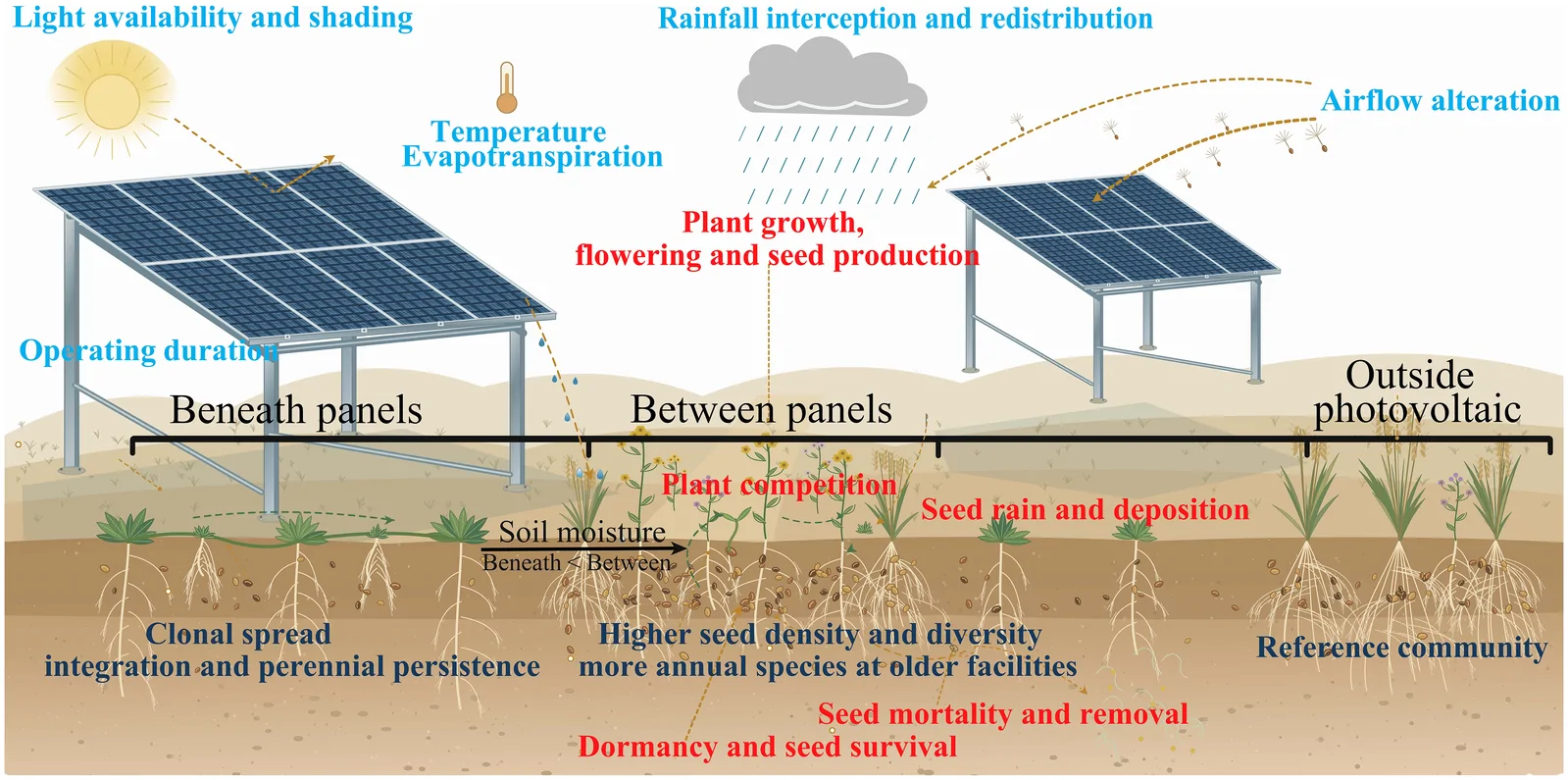
Potential mechanisms underlying the responses of aboveground vegetation and soil seed banks to microhabitat changes induced by photovoltaic installations in desert grasslands.

## 5 Conclusion

In contrast to previous studies based largely on remote sensing, this study provides direct field-based evidence of aboveground vegetation and soil seed bank responses to PV installations in desert grasslands. Comparisons among sites with different operating durations showed that soil seed bank density and α-diversity of both aboveground vegetation and soil seed banks increased between panels but decreased beneath panels. Meanwhile, dominant species contributed less to community differentiation at longer-operating sites, indicating more distributed species contributions. Trait-based analyses further revealed increasing proportions of perennial prostrate taproot clonal species beneath panels and annual species between panels across sites with longer operating durations. Together, these findings suggest that PV-associated microhabitat heterogeneity may influence plant community assembly and highlight the importance of microhabitat-specific vegetation management and ecological restoration in comparable desert grassland PV systems. Future studies should separate the effects of operating duration from those of PV configuration and investigate how variations in water and nutrient availability, together with microbial processes, influence soil seed bank dynamics and vegetation development.

## Data Availability

The original contributions presented in the study are included in the article/supplementary material. Further inquiries can be directed to the corresponding author.
