# Supplementary material for: Spatiotemporal patterns of aboveground vegetation and soil seed banks in desert steppe under photovoltaic disturbance
Source: Front Plant Sci. 2026 Oct 28;17:2000199. doi: 10.3389/fpls.2026.2000199 (PMC13751966; doi:10.3389/fpls.2026.2000199)
Supplement: Supplementary file 1 [file DataSheet1.docx]

**Spatiotemporal patterns of aboveground vegetation and soil seed banks in desert steppe under photovoltaic disturbance**

Qing Bai ^a1^, Hao Zhang ^b1^, Xiaomei Gong ^c^, Jianchu Xu ^d, e^, Qiang Yu ^b^, Changhui Peng ^f, g^, Xiaowei Li ^a^, Haiying Hu ^a^, Jianping Li ^a^, Fan Sang ^h^, Yingzhong Xie ^a *^, Liang Guo ^b, h, i*^

^a^ School of Forestry and Grassland Science, Ningxia University, Yinchuan, Ningxia 750000, China

^b^ State Key Laboratory of Soil and Water Conservation and Desertification Control, College of Soil and Water Conservation Science and Engineering, Northwest A&F University, Yangling, Shaanxi 712100, China

^c^ Ningxia International Desertification Control Cooperation Center, Yinchuan, Ningxia 750001, China

^d^ Center for Mountain Ecosystem Studies, Kunming Institute of Botany, Chinese Academy of Sciences, Kunming, Yunnan 650201, China

^e^ World Agroforestry Center, Nairobi 00100, Kenya

^f^ School of Geographic Sciences, Hunan Normal University, Changsha, Hunan 410081, China

^g^ Department of Biology Science, Institute of Environment Sciences, University of Quebec at Montreal, Montreal, QC H3C3P8, Canada

^h^ Administration Bureau of Ningxia Yunwushan National Nature Reserve, Guyuan, Ningxia 756000, China

^i^ Institute of Soil and Water Conservation, Chinese Academy of Sciences and Ministry of Water Resources, Yangling, Shaanxi 712100, China

*Correspondence: Yingzhong Xie, Liang Guo.

^1^: These authors contributed equally.

Email: xieyz@nxu.edu.cn, guoliang2014@nwafu.edu.cn

**Table S1.** Six plant functional traits used in the present study

| Traits | Type |
| --- | --- |
| Life form | Perennial, Annual |
| Growth form | Erect, Rosette, Prostrate |
| Root type | Taproot, Fibrous root, Axial root |
| Seed dispersal type | Gravity dispersal, Wind dispersal, Ballistic dispersal |
| Clumping Status | Clumping, Non-clumping |
| Root clonal ability | Clonally capable, Non-clonal |

**Table S2.** Species and family–genus composition identified from aboveground vegetation surveys and soil seed bank germination experiments.

| Species | Family | Genus |
| --- | --- | --- |
| *Stipa breviflora* | Poaceae | *Stipa* |
| *Lespedeza potaninii* | Fabaceae | *Lespedeza* |
| *Cleistogenes caespitosa* | Poaceae | *Cleistogenes* |
| *Peganum nigellastrum* | Nitrariaceae | *Peganum* |
| *Tournefortia sibirica* | Boraginaceae | *Tournefortia* |
| *Braya humilis* | Brassicaceae | *Neotorularia* |
| *Elymus dahuricus* | Poaceae | *Elymus* |
| *Convolvulus ammannii* | Convolvulaceae | *Convolvulus* |
| *Euphorbia humifusa* | Euphorbiaceae | *Euphorbia* |
| *Artemisia scoparia* | Asteraceae | *Artemisia* |
| *Setaria viridis* | Poaceae | *Setaria* |
| *Aster altaicus* | Asteraceae | *Heteropappus* |
| *Sophora alopecuroides* | Fabaceae | *Sophora* |
| *Oxytropis racemosa* | Amaranthaceae | *Obione* |
| *Corispermum mongolicum* | Amaranthaceae | *Corispermum* |
| *Olgaea leucophylla* | Asteraceae | *Olgaea* |
| *Grubovia dasyphylla* | Amaranthaceae | *Bassia* |
| *Plantago minuta* | Plantaginaceae | *Plantago* |
| *Artemisia blepharolepis* | Asteraceae | *Artemisia* |
| *Potentilla supina* | Rosaceae | *Potentilla* |
| *Teloxys aristata* | Amaranthaceae | *Dysphania* |
| *Limonium bicolor* | Plumbaginaceae | *Limonium* |
| *Lactuca tatarica* | Asteraceae | *Lactuca* |
| *Veronica undulata* | Plantaginaceae | *Veronica* |
| *Thermopsis lanceolata* | Fabaceae | *Thermopsis* |
| *Cynanchum chinense* | Apocynaceae | *Cynanchum* |
| *Eragrostis minor* | Poaceae | *Eragrostis* |
| *Portulaca oleracea* | Portulacaceae | *Portulaca* |
| *Polygala tenuifolia* | Polygalaceae | *Polygala* |
| *Salsola collina* | Amaranthaceae | *Salsola* |
| *Vincetoxicum mongolicum* | Apocynaceae | *Cynanchum* |
| *Tribulus terrestris* | Zygophyllaceae | *Tribulus* |
| *Stipa bungeana* | Poaceae | *Stipa* |
| *Gueldenstaedtia stenophylla* | Fabaceae | *Gueldenstaedtia* |
| *Convolvulus arvensis* | Convolvulaceae | *Convolvulus* |
| *Leymus secalinus* | Poaceae | *Leymus* |
| *Artemisia frigida* | Asteraceae | *Artemisia* |
| *Melilotus officinalis* | Fabaceae | *Melilotus* |
| *Oxytropis aciphylla* | Fabaceae | *Oxytropis* |

Note: Among these species, *Plantago minuta*, *Artemisia blepharolepis*, *Potentilla supina*, and *Teloxys aristata* were found exclusively in the soil seed bank.

**Table S3.** Soil seed bank density and proportional contribution across different soil layers.

| Treatment | 0-5 cm | 0-5/0-10 cm | 5-10 cm | 5-10/0-10 cm | Significance |
| --- | --- | --- | --- | --- | --- |
| 5 Outside | 1012.9±171.3 | 85.71% | 169.1±31.4 | 14.29% | ** |
| 5 Between | 1438.8±53.0 | 86.91% | 216.9±23.8 | 13.09% | *** |
| 5 Beneath | 517.4±183.4 | 79.08% | 63.7±11.3 | 10.05% | * |
| 10 Outside | 1038.8±193.3 | 87.72% | 143.3±22.5 | 12.28% | ** |
| 10 Between | 2051.7±95.6 | 90.06% | 226.9±37.6 | 9.94% | *** |
| 10 Beneath | 135.3±59.9 | 97.47% | 4.0±4.6 | 2.53% | * |
| 15 Outside | 1118.4±126.3 | 89.30% | 133.3±7.6 | 10.70% | *** |
| 15 Between | 2596.9±66.1 | 90.20% | 282.6±29.4 | 9.80% | *** |
| 15 Beneath | 81.6±39.7 | 98.44% | 2.0±4.0 | 1.56% | * |

Note: Data are presented as mean ± standard deviation (SD). “Outside” refers to areas outside the photovoltaic (PV) field, while “Between” and “Beneath” represent locations between and beneath PV panels, respectively. The numbers5, 10, and 15 represent PV operational durations in years. Asterisks (*, **, ***) indicate significant differences in soil seed bank density between the 0–5 cm and 5–10 cm soil layers: *P* < 0.05, *P* < 0.01, and *P* < 0.001, respectively.

**Table S4.** Soil seed bank density in PV stations of different operational durations.

| Treatment | 5 Year | 10 Year | 15 Year |
| --- | --- | --- | --- |
| Outside | 1182.06 ± 101.18 | 1182.06 ± 101.34 | 1251.71 ± 66.87 |
| Between | 1656.68 ± 30.96 | 2278.55 ± 56.41 | 2879.53 ± 46.11 |
| Beneath | 641.78 ± 32.90 | 139.30 ± 31.42 | 83.58 ± 39.80 |

Note: Data are presented as mean ± standard deviation (SD). “Outside” refers to areas outside the photovoltaic (PV) field, while “Between” and “Beneath” represent locations between and beneath PV panels, respectively. The numbers5, 10, and 15 represent PV operational durations in years. The unit for soil seed bank density is seeds m⁻².

**Fig. S1.** Annual precipitation and mean annual temperature at each of the three photovoltaic (PV) sites from 2010 to 2024.

**Fig. S2.** Assessment of climatic equivalence across the three photovoltaic (PV) stations with different operational durations. (a) Annual mean temperature, 1901–2024. (b) Annual precipitation, 1901–2024. (c) Relationship between annual mean temperature at the 5-year and 15-year sites. (d) Relationship between annual precipitation at the 5-year and 15-year sites. Pearson’s r indicates the synchrony of interannual fluctuations in temperature and precipitation between the two sites; Site var (site variance, ANOVA) represents the proportion of total climate variance attributable to site identity rather than to interannual variation; and ICC site (intraclass correlation coefficient) denotes the degree to which the three sites are climatically interchangeable, with values near zero indicating interchangeability.

**Fig. S3.** Relative changes in genus-level species richness of aboveground vegetation across different microhabitats within photovoltaic (PV) stations of different operational durations. “Outside” indicates areas outside the PV field, while “Between” and “Beneath” denote locations between and beneath PV panels, respectively. The numbers 5, 10, and 15 represent PV operational durations in years. The same notation applies to subsequent figures.

**Fig. S4.** Relative changes in genus-level species richness of soil seed banks across different microhabitats within photovoltaic (PV) stations of different operational durations.

**Fig. S5.** NMDS ordination (a, c, e) and SIMPER contribution analysis (b, d, f) of aboveground vegetation composition across different photovoltaic (PV) microhabitats. Asterisks (*) indicate significant differences between groups along the NMDS axes.

**Fig. S6.** Microclimatic conditions across different microhabitats within photovoltaic (PV) stations with the same operational duration. Different lowercase letters (a, b, and c) indicate significant differences among microhabitats within the same PV operational duration (*P* < 0.05).
